# Effect of pharmacogenomics testing guiding on clinical outcomes in major depressive disorder: a systematic review and meta-analysis of RCT

**DOI:** 10.1186/s12888-023-04756-2

**Published:** 2023-05-12

**Authors:** Xinrui Wang, Chenfei Wang, Yi Zhang, Zhuoling An

**Affiliations:** Department of Pharmacy, Beijing Chao-Yang Hospital, Capital Medical University, No. 8 Gongtinan Road, Chaoyang District, Beijing, 100020 China

**Keywords:** Pharmacogenomic, Major depressive disorder, Guided

## Abstract

**Background:**

Pharmacogenomic testing guided treatment have been developed to guide drug selection or conversion in major depressive disorder patients. Whether patients benefit from pharmacogenetic testing remains unclear. We aim to evaluates the effect of pharmacogenomic testing guiding on clinical outcomes of major depressive disorder.

**Methods:**

Pubmed, Embase, and Cochrane Library of Clinical Trials were searched from inception until August 2022. Key terms included pharmacogenomic and antidepressive. Odds ratios (RR) with 95% confidence intervals (95%CIs) were calculated using fixed-effects model for low or moderate heterogeneity or random-effects model for high heterogeneity.

**Results:**

Eleven studies (5347 patients) were included. Compared with usual group, pharmacogenomic testing guided group was associated with an increased response rate at week 8 (OR 1.32, 95%CI 1.15–1.53, 8 studies, 4328 participants) and week 12 (OR 1.36, 95%CI 1.15–1.62, 4 studies, 2814 participants). Similarly, guided group was associated with an increased rate of remission at week 8 (OR 1.58, 95%CI 1.31–1.92, 8 studies, 3971 participants) and week 12 (OR 2.23, 95%CI 1.23–4.04, 5 studies, 2664 participants). However, no significant differences were found between the two groups in response rate at week 4 (OR 1.12, 95%CI 0.89–1.41, 2 studies, 2261 participants) and week 24 (OR 1.16, 95%CI 0.96–1.41, 2 studies, 2252 participants), and remission rate at week 4 (OR 1.26, 95%CI 0.93–1.72, 2 studies, 2261 participants) and week 24 (OR 1.06, 95%CI 0.83–1.34, 2 studies, 2252 participants). Medication congruence in 30 days was significantly reduced in the pharmacogenomic guided group compared with the usual care group (OR 2.07, 95%CI 1.69–2.54, 3 studies, 2862 participants). We found significant differences between subgroups of target population in response and remission rate.

**Conclusion:**

Patients with major depressive disorder may benefit from pharmacogenomic testing guided treatment by achieving target response and remission rates more quickly.

**Supplementary Information:**

The online version contains supplementary material available at 10.1186/s12888-023-04756-2.

## Introduction

Major depressive disorder (MDD) is one of the most prevalent psychiatric diseases, affecting more than 300 million people globally [1]. MDD leads to cognitive impairment, which results in suicidal behaviors [2–5]. More than 800,000 people die from suicide each year [6]. For decades, medications for MDD followed clinical guidelines of MDD treatment, while numerous anti-depressant drugs went on the market and were considered to be “first-line” for comparable remission and/or response rates [7]. However, following the present anti-depressant treatment pathway, only 50% of patients achieve clinically significant responses within their first anti-depressant treatment; the number of patients achieving remission is even fewer [8]. Thus, the current standard treatment pathway is a trial-and-error approach until a relatively effective anti-depressant or combination treatment is found that provides full remission effects [7]. Current treatment approaches result in a prolonged duration of unremitted illness, which is related to worsened long-term prognosis, adverse changes in cognitive function and brain morphology, and increased side effects [9, 10]. Because of those problems with current MDD medications, better tools are urgently needed to help physicians and patients find effective treatment.

Pharmacogenomic testing offers a point-of-care, personalized, and ascendable tactic to guide clinical treatment in multiple diseases pre-emptively, including MDD [11]. A number of proprietary pharmacogenomic testing tools have been developed to guide drug selection or conversion by testing allelic gene variants of assigned genes mediating the pharmacodynamics and/or pharmacokinetics of anti-depressant medication [12]. Numerous randomized controlled trials (RCTs) have been conducted to evaluate the efficacy and safety outcomes of pharmacogenomic testing in MDD, but the results and conclusions are still conflicting. Several large-scale RCTs depicted significant improvement in response and remission rates, while a recent study by Oslin et al. showed no significant change between the pharmacogenetic testing guided treatment group and the usual treated group [13–16]. The key point of whether patients benefit from pharmacogenetic testing remains unclear.

Several previous systematic review and meta-analyses had been conducted based on this issue [12, 17, 18]. However, since the number of studies involved was limited, no sufficient data was provided to reach a reliable conclusion. On the other hand, cohort studies and RCTs were both included in the meta-analyses which could contribute to potential bias. Furthermore, plenty of RCTs regarding this topic have been published in recent two years [14, 15, 19], reflecting the necessity of an updated systematic review and meta-analysis. Here in our current study, we determined the effect of pharmacogenetic testing guided on clinical outcomes in MDD patients.

## Methods

### Protocol and registration

This is a protocol-driven systematic review and meta-analysis, prospectively registered with the International Prospective Register of Systematic Reviews (PROSPERO CRD42022360151). We performed this meta-analysis in accordance with the guideline of Preferred Reporting Items for Systematic reviews and Meta-Analyses (PRISMA) guideline [20], and the checklist is available in Supplemental Material (Method S1).

### Literature research

We searched PubMed, Embase, and Cochrane Library of Clinical Trials from inception until August 2022. The key terms were ‘pharmacogenomic’ and ‘antidepressive’. The detail of the search terms is attached in Table S1. The reference lists of all included studies were also examined for relevant citations. No language restrictions were applied.

### Study selection

Studies were considered for inclusion based on the following criteria: [1] participants were diagnosed with major depressive disorder; [2] the study included both pharmacogenomic testing guided group and usual care group; [3] randomized controlled trial (RCT). The screening and scanning for eligibility were performed manually by two independent reviewers (X. W. and C. W.) through Endnote (version 9.3.2). Any disagreements were resolved by discussion with a third reviewer (Z. A.).

### Quality assessment

Six domains (random sequence generation, allocation concealment, blinding of participants and personnel, blinding of outcome assessment, incomplete outcome data, and selective reporting) were assessed by using the Cochrane Collaboration’s tool for assessing the risk of bias [21] by two reviewers (X. W. and C. W.) independently. Disagreements on study quality assessment were discussed with another reviewer (Y. Z.) until a consensus was reached.

### Data extraction

Data were independently extracted by two investigators (X. W. and C. W.) for eligible studies. The characteristic data obtained for each study included the first author, year of publication, study design, inclusion criteria, target genes, baseline characteristics, and industry funding. Outcomes were response (≥ 50% decrease in HAM-D17/PHQ-9 score from baseline) rate and remission (a score of ≤ 7 for HAM-D17 or ≤ 5 for PHQ-9 or ≤ 2 for CGI score) rate at week 4, 8, 12, and 24, respectively. Another outcome was medication congruence in 30 days (participants that prescribed antidepressant medication that was categorized as having no drug-gene interaction or moderate drug-gene interaction). We gave preference to data from intent-to-treat analysis over pre-protocol analysis. Data with longer follow-up duration were chosen over the shorter one when data at several time points were provided within the period.

### Statistical analysis

Odds ratios (RR) with 95% confidence intervals (95%CIs) were calculated with R (version 4.0.5). Heterogeneity across the studies was assessed using the Cochran’s Q test; the percentage of total variability attributable to heterogeneity was quantified by the I^2^ value. I^2^ less than 50% indicates low or moderate heterogeneity and more than 50% indicates high heterogeneity [21]. Random-effects model with inverse variance weighting was used for high heterogeneity, and the Mantel-Haenszel fixed-effects model was used for low or moderate heterogeneity of included studies. P values less than 0.05 were considered significant. Funnel plots were used to test the publication bias.

Subgroup analysis was done according to follow-up duration for less than 12 weeks (data with the longest follow-up duration through the period was extracted). Only a few studies included provided data with longer duration, so subgroup analysis for outcomes at week 24 was not conducted. Four further subgroup analyses were carried out according to study design (single-center or multi-center), sample size (less than 200 patients or more than 200 patients), population (the majority ethnicity of the population: Caucasian or Asian), diagnostic criteria (HAM-D17 or PHQ-9), and industry funding (fully funded, partially funded, or none funded). A difference between the estimates of these subgroups was considered significant for the P-value between subgroups < 0.10 [22].

To evaluate the stability of the results, we did a sensitivity analysis by sequentially removing every single study from the pooled effect estimates.

## Results

1779 potentially relevant articles were screened. 1712 irrelevant articles were excluded based on screening of the titles and abstracts. 54 articles went through full-text review and 43 of them were excluded because they did not meet the inclusion criteria. Lastly, eleven RCT studies with a total of 5347 patients were included in the meta-analysis (Figure S1). The duration of follow-up was 8 weeks in four studies, 10 weeks in one study, 12 weeks in three studies, 24 weeks in two studies, and 52 weeks in one study. The specific genes tested differed between studies (Table 1).

**Table 1:** Baseline characteristics of the randomized controlled studies assessing the effect of pharmacogenomics testing guided in major depressive disorder patients

Study	Year	Study design	Inclusion	Gene testing system	Molecular method	Target genes	Baseline characteristics	Target population	Industry funding
Bradley	2017	12-week, prospective, subject- and rater-blind, multi-center	·age 19–87 ·dianosed with depression and /or anxiety using the DSM-5 criteria of standard of care site procedures and verified by the MINI Psychiatic Interview ·HAM-D17 ≥ 18	NeuroIDgenetix	End-point PCR, real-time PCR, capillary electrophoresis	CYP1A2, CYP2C9, CYP2C19, CYP2D6, CYP3A4, CYP3A5, SLC6A4, [NM_001045.5:c.-1760 C > T], SLC6A4 [5-HTTLPR], COMT [NM_000754.3:c.472G > A], HTR2A [NM_000621.4:c.-998G > A], HTR2A [NM_000621.4:c.614e2211T > C], MTHFR [NM_005957.4:c.665 C > T], MTHFR [NM_005057.3:c.1286 A > C]	Guided group (n = 352): age 47.8 ± 14.5; female 73%; Usual group (n = 333): age 47.3 ± 15.2; female 72%	Caucasian 63%, African-American 18%, Hispanic 17%, Asian 1%, other 1%	Fully
Greden	2019	24-week, prospective, subject- and rater-blind, multi-center	·age > 18 ·diagnosed with MDD (≥ 11 on the QIDS-C16 and self-rated QIDS-SR16 at screening and baseline) ·had an inadequate responseto at least one documented psychotropic treatment included on thepharmacogenomic test report within the current depressive episode	GeneSight	NA	CYP1A2, CYP2C9, CYP2C19, CYP3A4, CYP2B6, CYP2D6, HTR2A, SLC6A4	Guided group (n = 681): age 46.9 ± 14.5, female 71.8%; Usual group (n = 717): age 48 ± 14.5, female 69.5%	White 80.6%, Black 14.9%, Asian 2.1%, other 2.4%	Partially
Han	2018	8-week, subject-blind, multi-center	·age ≥ 20 ·diagnosed with MDD according to DSM-5 criteria ·showed 3 or more on CGI-Improvement score despite of current antidepressant treatment with roper dosage at least 6 weeks or intolerance to current anti-depressant therapy based on clinicians’ judgement.	Neuropharmagen	NA	NA	Guided group (n = 52): age 44.2 ± 16.1, female 76.9%; Usual group (n = 48): age 43.9 ± 13.8, female proportion 72.9%	Asian 100%	Partially
McCarthy	2021	8-week, prospective, subject-blind, multi-center	·current depression in the context of Stage 1 or higher treatment-resistant depression	TaqMan-PCR (Pathway Genomics)	TaqMan-PCR	CYP1A2, CYP2C9, CYP2C19, CYP3A4, CYP3A5, CYP2B6, CYP2D6, HTR2A, SLC6A4, DRD2, HLARegion, 5HTTLPR, HTR2A, HTR2C, POLG, UGT1A4	Guided group (n = 75): age mean ± SEM 52.5 ± 1.5, female 21%; Usual group (n = 74): age 50.3 ± 1.6, female 26%	NA	Partially
Oslin	2022	24-week, prospective, rater-blind, pragmatic, multi-center	·receiving care at VA medical centers ·aged 18–80 ·with a diagnosis of MDD ·a history of at least 1 treatment episode ·a plan to start a new episode of antidepressant monotherapy ·PHQ-9 > 9	GeneSight	NA	CYP1A, CYP2B6, CYP2C19, CYP2C9, CYP3A4, CYP2D6, UGT1A4, UGT2B15, SLC6A4, HTR2A, HLA-B*1502, HLA-A*3101	Guided group (n = 966): age 48 ± 15, female 24%; Usual group (n = 978): age 47 ± 15, female 27%	White 69%, African American 18%, Asian Pacific Islander 3%, Native American 1%, other 9%	Partially
Perez	2017	12-week, prospective, subject- and rater- blind, multi-center	·age ≥ 18 ·with a principal diagnosis of MDD ·subjects with a clinician rated score in the CGI-S scale ≥ 4 ·required medication de novo or were receiving treatment and required substitution or addition of drug treatment with an antidepressant drug	OpenArray	Real-time PCR, TaqMan-PCR	ABCB1, AKT1, BDNF, CACNG2, CES1, COMT, CRHR1, CYP1A2, CYP2B6, CYP2C19, CYP2C9, CYP2D6, CYP3A4, DDIT4, DRD3, EPHX1, FCHSD1, GRIK2, GRIK4, HLA-A, HTR1A, HTR2A, HTR2C, LPHN3, NEFM, OPRM1, RGS4, RPTOR, SLC6A4, UGT2B15	Guided group (n = 155): age 51.74 ± 12.05, female 63.9%; Usual group (n = 161): age 50.74 ± 13.12, female 63.4%	Caucasian 92.40%, Latin American 5.38%, other 2.22%	Partially
Perlis	2020	8-week, prospective, subject- and rater-blind, multi-center	·age 18–75 ·with a primary diagnosis of nonpsychotic MDD based on DSM-5 criteria and MINI7.0, and HAM-D17 score > 18 ·have failure of at least one prior adequate trial of a standard antidepressant for the current major depressive episode	Genecept Assay	NA	45 variants of 7 pharmacokinetic cytochrome P450 genes and 12 variants of 11 pharmacodynamic or other genes	Guided (n = 151): age 47.8 ± 12.38, female 70.9%; Usual (n = 153): age 47.6 ± 12.06, female 72.5%	White 72.7%, African American 23.4%, Asian 0.3%, Native American 1%, Pacific Islander 1%, other 1.6%	Fully
Shan	2019	8-week, prospective, subject-blind, single-center	·age 18–51 ·at least a junior high school education level with the ability to understand survey contents ·HAM-D17 score ≥ 17 at baseline and the first item of the HAM-D17 (depressive mood) ≥ 2 ·never received psychiatric treatment or have interrupted antidepressant medication for more than 2 weeks (fluoxetine for at least 4 weeks) ·with no psychotic symptoms	TaqMan probe–PCR MassARRAY DNA	TaqMan probe-PCR, MALDI-TOF mass spectrometry	CYP2C19, CYP2D6, CYP1A2, SLC6A4, 5-HTR2A	Guided group (n = 31): age 26.52 ± 7.92, female proportion 19; Unguided group (n = 40): age 28.85 ± 8.93, female 65%	Asian	None
Singh	2015	12-week, perspective, subject- and rater- blinded, single-center	·with a principal DSM-5 diagnosis of MDD ·HAM-D17 score > 18	CNSDose	PCR, MALDI-TOF mass spectrometry	ABCB1, ABCC1, CYP2D6, CYP2C19	Guided group (n = 74); mean age 44.2, male 42%; Usual group (n = 74); mean age 44.3, male 39%	NA	Fully
Tiwari	2022	52-week, prospective, patient- and rater-blinded, multi-center*	·age ≥ 18 ·diagnosed with MDD according to DSM-4 criteria, QIDS-C16 score ≥ 11 at screening and baseline ·had inadequate response to at least one psychotropic medication included on the combinatorial pharmacogenomic report within the current depressive episode	GeneSight Enhanced GeneSight	PCR, gel electrophoresis, xTAG assay	CYP1A2, CYP2B6, CYP2C9, CYP2C19, CYP2D6, CYP3A4, HTR2A, SLC6A4, MC4R, CNR1, NPY, GCG, HCRTR2, NDUFS1	Guided group (n = 90): age 40.3 ± 15.3, female 65.6%; Usual group (n = 93): age 42.3 ± 14.2, female 63.4%	Caucasian 84.1%, Asian 8.7%, Black 2.9%, Latin American 1.8%, other 2.5%	Partially
Winner	2013	10-week, prospective subject-and rater-blind, single-center	·with a diagnosis of MDD or DDNOS were approached for enrollment by their treating clinician ·HAMD-17 score ≥ 14	GeneSight Luminex xTAG PCR	PCR, gel electrophoresis, xTAG assay	CYP2D6, CYP2C19, CYP1A2, SLC6A4, HTR2A	Guided group (n = 25): age 50.6 ± 14.6. female 69%; Usual group (n = 24): age 47.8 ± 13.9. female 92%	Non-Hispanic White 98%, Black 2%	Fully

MDD, major depressive disorder; HAM-D17, 17-item Hamilton Depression Rating Scale; QIDS-C16, 16-item Quick Inventory of Depressive Symptomatology-Clinician Rated; NA, not applicable; QIDS-SR16, 16-item Quick Inventory of Depressive Symptomatology- Self-Report; DSM-5, Diagnostic and Statistical Manual of Mental Disorders, 5th Edition; SD, bipolar disorder; PTSD, post-traumatic stress disorder; PHQ-9, 9-item Patient Health Questionnaire; SATMED-Q, Treatment Satisfaction with Medicines Questionnaire; CGI-S, Clinical Global Impression-Severity; DDNOS, depressive disorder not otherwise specified; *data available only until week 24

### Assessment of risk

The overall result of the risk assessment is summarized in Figure S2. Most of the studies were categorized as having a high bias in the other bias section since most of them were funded by industry. Only one study [23] received no industry funding. One study [15] was assigned high bias in the blinding of participants and personnel section since it was a single-blinded (rater-blinded) study; while others that assigned low bias were double-blind (patient- and rater-blinded) studies. Funnel plots of outcomes were presented in Figure S3.

### Response rate

**Fig. 1:**
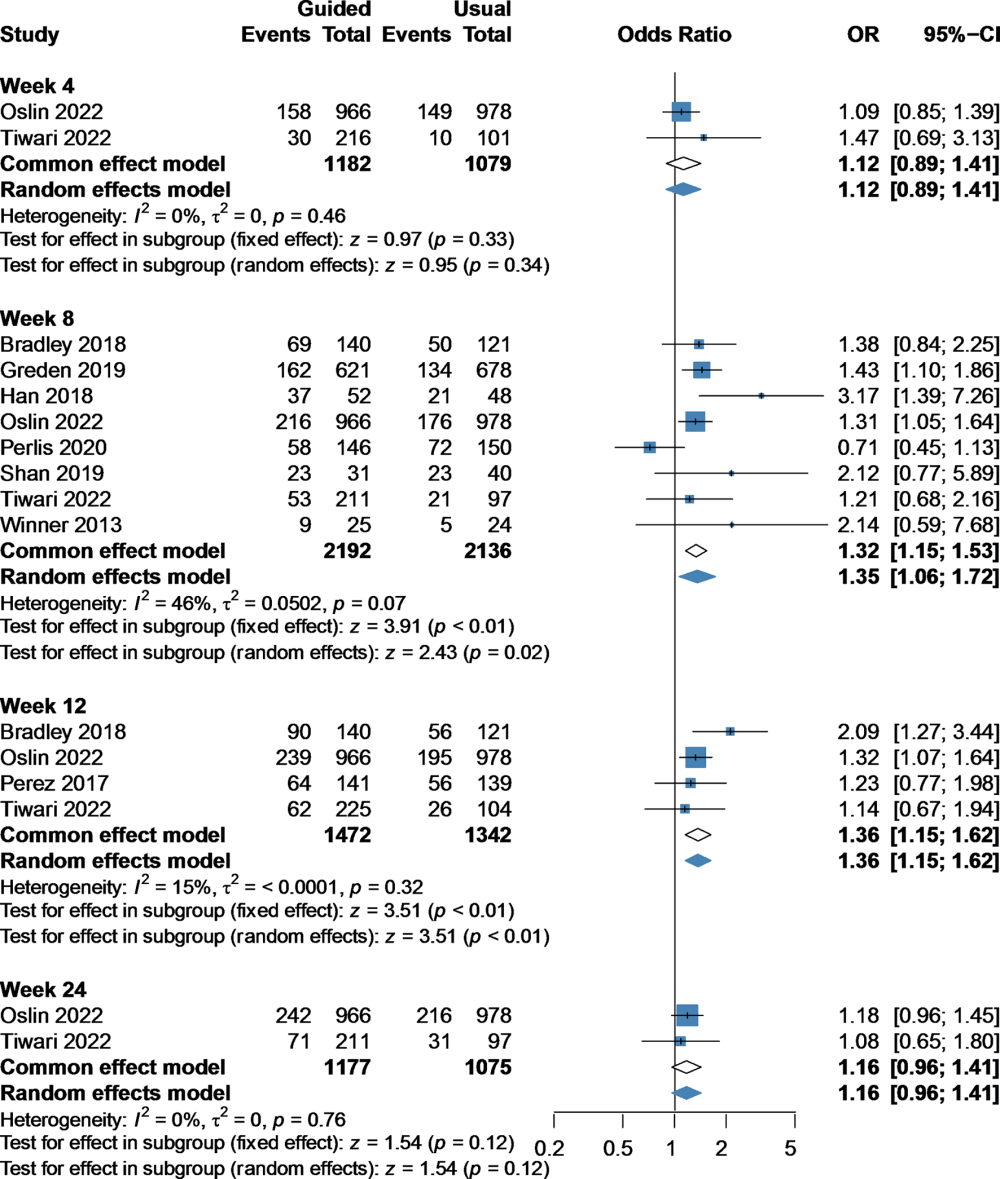
Forest plot of response rate at week 4, 8, 12, and 24 comparing pharmacogenomic testing guided treatment versus usual care treatment. OR, odds ratio; CI, confidence interval

Response rates were significantly increased in the guided group compared with the usual group at week 8 (OR 1.32, 95%CI 1.15–1.53, 8 studies, 4328 participants) [13–16, 23–26] and week 12 (OR 1.36, 95%CI 1.15–1.62, 4 studies, 2814 participants) [14, 15, 24, 27]. However, no significant difference was observed between the guided group and the usual group at week 4 (OR 1.12, 95%CI 0.89–1.41, 2 studies, 2261 participants) [14, 15] or week 24 (OR 1.16, 95%CI 0.96–1.41, 2 studies, 2252 participants) [14, 15] (Fig. 1).

### Remission rate

**Fig. 2:**
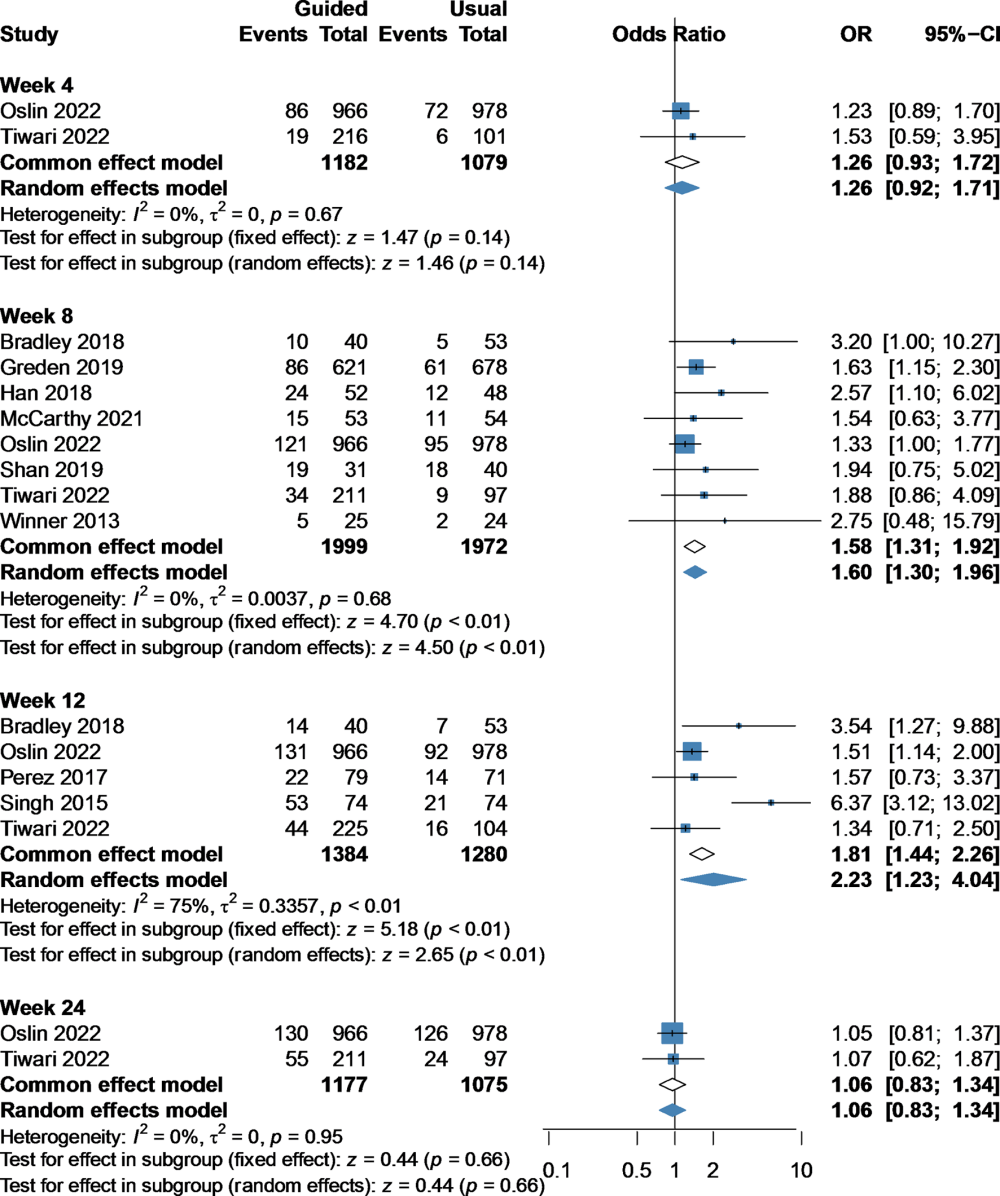
Forest plot of remission rate at week 4, 8, 12, and 24 comparing pharmacogenomic testing guided treatment versus usual care treatment. OR, odds ratio; CI, confidence interval

Compared with the usual group, the guided group was associated with an increased remission rate at week 8 (OR 1.58, 95%CI 1.31–1.92, 8 studies, 3971 participants) [13–15, 19, 23–26] and week 12 (OR 2.23, 95%CI 1.23–4.04, 5 studies, 2664 participants) [14, 15, 24, 27, 28]. Similarly, we found no significant difference between the guided group and the usual group at week 4 (OR 1.26, 95%CI 0.93–1.72, 2 studies, 2261 participants) [14, 15] and week 24 (OR 1.06, 95%CI 0.83–1.34, 2 studies, 2252 participants) [14, 15] (Fig. 2).

### Medication congruence in 30 days

**Fig. 3:**
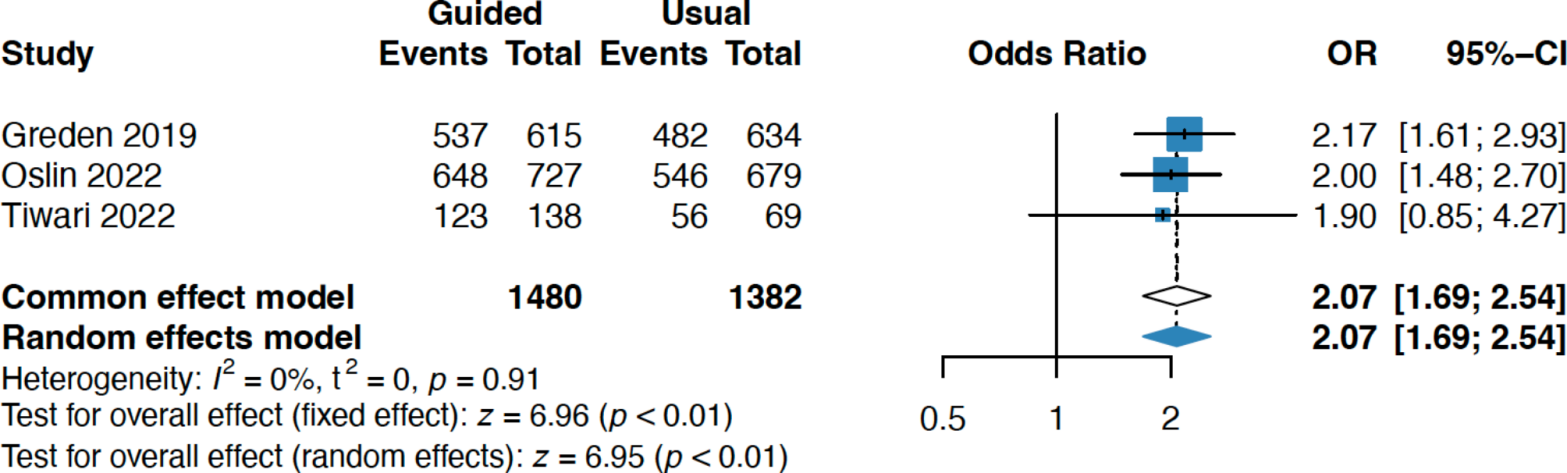
Forest plot of medication congruence in 30 days comparing pharmacogenomic testing guided treatment versus usual care treatment. OR, odds ratio; CI, confidence interval

Medication congruency in 30 days was significantly reduced in the pharmacogenomic guided group compared with the usual care group (OR 2.07, 95%CI 1.69–2.54, 3 studies, 2862 participants) [13–15](Fig. 3).

### Subgroup analysis and sensitivity analysis

Table 2 shows the subgroup analysis according to several parameters. Specifically, we found significant differences between subgroups of the target population in both response (P-value between subgroup = 0.03) and remission rate (P-value between subgroup < 0.01). In addition, significant differences between subgroup in subgroup analyses according to study design, sample size, and industry funding of remission rate were also observed, while no significant difference between subgroups was found between different diagnostic criteria groups; however, the pharmacogenomic testing guided group compared with usual group were all associated with an increased rate of remission in subgroups.

**Table 2:** Subgroup analysis of remission and response rate in week 0–12 in studies comparing pharmacogenomics testing guided and usual care group in MDD patients

		Response				Remission			
		Study /sample size	OR (95%CI)	I_2_ (%)	P_between_	Study /sample size	OR (95%CI)	I_2_ (%)	P_between_
Study design	Single-center	2/120	2.13 (0.96, 4.73)	0	0.26	3/268	**3.96 (2.32, 6.78)**	**50**	**< 0.01**
	Multi-center	7/4509	**1.34 (1.04,1.77)**	**60**		7/4022	**1.62 (1.34, 1.95)**	**0**	
Sample size	< 200	4/549	**1.66 (1.13, 2.45)**	**35**	0.27	6/804	**2.36 (1.37, 4.05)**	**57**	**0.07**
	≥ 200	5/4080	1.29 (0.97, 1.72)	62		4/3486	**1.61 (1.31, 1.98)**	**0**	
Population	Caucasian	7/4458	**1.31 (1.14, 1.50)**	**47**	**0.03**	7/3971	**1.59 (1.31, 1.92)**	**0**	**< 0.01**
	Asian	2/171	**2.70 (1.42, 5.13)**	**0**		2/171	**2.27 (1.20, 4.28)**	**0**	
	NA	-	-	-		1/107	-	-	
Diagnostic criteria	HAM-D17	8/2555	**1.57 (1.11, 2.23)**	**60**	0.48	9/2535	**1.95 (1.24, 3.07)**	**71**	0.46
	PHQ-9	2/3243	**1.37 (1.17, 1.60)**	**0**		2/3243	**1.42 (1.16, 1.74)**	**0**	
Industry funding	Fully	3/606	1.37 (0.62, 3.00)	81	0.70	3/290	**4.89 (2.81, 8.52)**	**0**	**< 0.01**
	Partially	5/3952	**1.37 (1.18, 1.59)**	**17**		6/3929	**1.57 (1.30, 1.90)**	**0**	
	None	1/71	-	-		1/71	-	-	

MDD: major depressive disorder; OR, odds ratio; CI, Confidence interval; P_between_, P-value between subgroup; NA, not available; HAM-D17, 17-item Hamilton Depression Rating Scale; PHQ-9, 9-item Patient Health Questionnaire; **bold** indicate significant difference

No outcome changed when performing sensitivity analysis using the leave-one-out method (Figure S4).

## Discussion

The current meta-analysis identified 11 RCTs that compared the effect of pharmacogenomic testing on target outcomes were identified. Quantitative pooled analysis showed that pharmacogenomic testing guided treatment contributed to improved response and remission rates compared to usual treatment at week 8 and 12, while no significantly higher response or remission rates were found at week 4 or 24. Pharmacogenomic testing guided treatment shortened the time to clinical remission and response to antidepressants. In addition, the pharmacogenomic testing guided treatment decreased medication congruence compared to usual care. In subgroup analysis, we found significant differences between the Asian subgroup and the Caucasian subgroup.

Overall, these data showed that pharmacogenomic testing guided treatment helped MDD patients achieve clinical remission or response in a shorter time compared to usual treatment while making no difference in final response or remission rates at the end of the pharmacogenomic testing guided treatment. Notably, our results were different from previous meta-analyses, which simply showed higher response and remission rates in pharmacogenomic testing guided MDD patients than usual treated patients [12, 18, 29]. When combining all the outcomes at any follow-up duration from included studies, we found similar results as the previous studies (Figure S5). However, when separating the data into week 4, 8, 12, and 24, we found significantly improved response and remission rates in week 8 and 12 rather than 4 and 24. No significant changes were found at week 4 may because of the long onset time of anti-depressants, and no significant changes were found at week 24 may because pharmacogenomic testing guidance only showed a ‘catalyst-like’ effect by accelerating the process of excluding the unsuitable anti-depressants for MDD patients rather than improving the MDD therapeutic effects.

This current meta-analysis found fewer gene-drug interactions in MDD patients receiving pharmacogenomic testing guided treatment than usual in those receiving usual treatment. These results showed an effect of pharmacogenomic testing guidance on decreasing severe adverse effects induced by gene-drug interactions during MDD medications. Pharmacogenomic testing guidance provided relevant clinical information on the effect of an anti-depressant, potential gene-drug interactions, and adverse effects for physicians and patients, helping to make more accurate decisions [15].

The genes that encode hepatic cytochrome P450 (CYP450) enzymes receive great concerns in pharmacogenomic testing, because of their roles in the regulations of psychotropic drug metabolism [30]. Previous studies revealed the critical role of CYP450 enzymes on the pharmacokinetics of anti-depressants, especially CYP2C19 and CYP2D6 [31, 32], with high-level evidence showing relevance to medications commonly used in psychiatry practice [32–34]. Briefly, findings showed that variants of CYP2C19 and CYP2D6 have been associated with blood concentrations of antidepressants, adverse drug reactions, and clinical outcomes of antidepressants [35, 36]. Moreover, variations of other genes were also thought to be closely associated to different responses to antidepressants, such as the human gene-encoding serotonin transporter (SCL6A4) gene, catechol-o-methyltransferase (COMT) gene, and serotonin-2 A receptor (HTR2A) gene [37–40].

In subgroup analysis, the current study showed a significant difference between the Asian subgroup and the Caucasian subgroup. The difference may be associated with the sub-genotype of allele frequencies of the gene variants. For example, more CYP2C19 and CYP2D6 variants were found in Asian people than in Caucasians. Almost 20% of Asians were CYP2C19 poor metabolizers while 3% of Caucasians were poor metabolizers [41, 42]; only 1% of healthy Asians were attributed to poor metabolizers of CYP2D6 while the proportion of Caucasians was 5–10% [43, 44]. Furthermore, allele frequencies of the SLC6A4 gene variant between Caucasians and Asians are different, the S allele being present in 42% of Caucasians but in 79% of Asians [45]. The difference in pharmacokinetics enzymes led to different responses to anti-depressants between Asian and Caucasian. Among those genes which were reported to have different responses to antidepressants, CYP450 enzyme genes, especially CYP2C19 and CYP2D6, may mask the effects of other genes because of the sensible effects on the regulation of antidepressant metabolism.

But meanwhile, it’s still challenging for pharmacogenomic testing guidance utilized in clinical MDD medications. Firstly, there is not enough evidence to support the use of pharmacogenomic testing guidelines in MDD clinical practice. The US Food and Drug Administration cautioned about the content validity and potentially detrimental impact of pharmacogenomic testing panels, while Dutch and French guidelines recommended pharmacogenomic testing guided treatment as a potentially useful tool of MDD medication [46, 47]. Although the current study showed the effects of pharmacogenomic testing guidance on shortening the time to identify an appropriate antidepressant, there are still difficulties in developing pharmacogenomic testing guidance on MDD medications. Secondly, the cost-effectiveness of pharmacogenomic testing guidance remains unclear. The average number of genes tested in studies included in the current meta-analysis was more than 10, which may lead to huge expenditure and result in low acceptance of MDD patients. Though pharmacogenomic testing may not lead to less direct cost than usual antidepressant treatment, it’s still a very profitable tool to decrease taking inappropriate antidepressants of MDD patients and help reduce indirect costs [48–50]. Moreover, several institutions provide different pharmacogenomic testing tools and services; all those tools have their unique pipelines, and results are not provided uniformly [51, 52]. Thus, clinicians should get support from genetic counselors, pharmacists, or companies for proper implementation [29]. Previous case reports highlighted the key role of pharmacist assessment during drug-gene interactions and drug‐induced pheno-conversion in MDD. Further open-label RCT about pharmacist-guided pharmacogenetic testing in antidepressant therapy is in progress [53, 54]. Pharmacogenomic testing guidance would make a huge difference in MDD medications if those problems could be resolved.

The current study included only RCTs with longer follow-up duration and a much larger sample size. We also analyzed data at multiple time points and conducted multiple comprehensive sub-group analyses. Although this study revealed the effect of pharmacogenomic testing guided treatment on MDD medications and indicated its striking value in Asian MDD patients, there are still several methodological limitations that may lead to interference and bias that emerged from this study. The reliability of the inclusion of unblinded clinicians was reduced in most of the reported pooled effect studies. In addition, patients were unblinded in the largest scale research included in this current analysis, which may also lead to detection bias of outcomes. Another significant limitation of this current study is on basis of data in per-protocol analysis rather than intention-to-treat analysis, which may lead to overestimating the outcomes of pharmacogenomic testing guided treatment on MDD medications. Nevertheless, this meta-analysis is a re-analysis based on current research so a few genotypes were involved. Previous reports focused on the effects of CYP2C19 and CYP2D6 on antidepressants, thus our research mostly mentioned the two variants [31, 32]. In the future, more genotypes would be involved in our prospective research.

## Conclusions

Our study demonstrated the effect of pharmacogenomic testing guidance in shortening the process of reaching clinical remission and response to anti-depressant medications in MDD. Future well-designed multi-ethnic studies are needed to confirm the benefit of pharmacogenomic testing guidance in different populations.

## Electronic supplementary material

Below is the link to the electronic supplementary material.

**Supplementary Material 1 Figure S1**. PRISMA Flow Diagram

**Supplementary Material 2 Figure S2**. Summary of Risk Assessment Using the Cochrane Collaboration’s Tool For Assessing the Risk of Bias

**Supplementary Material 3 Figure S3**. Funnel plot of outcomes

**Supplementary Material 4 Figure S4**. Sensitivity Analysis of included studies

**Supplementary Material 5 Figure S5**. Forest plot of response and remission rate comparing pharmacogenomic guided treatment versus usual care treatment when not separating data into different weeks

**Supplementary Material 6 Table S1**. Search Terms

## Data Availability

The datasets used and/or analyzed during the current study are available from the corresponding author upon reasonable request.
