# Supplementary material for: Effect of pharmacogenomics testing guiding on clinical outcomes in major depressive disorder: a systematic review and meta-analysis of RCT
Source: BMC Psychiatry. 2023 May 12;23:334. doi: 10.1186/s12888-023-04756-2 (PMC10176803; doi:10.1186/s12888-023-04756-2)
Supplement: Supplementary file 1 — Supplementary Material 1 Figure S1. PRISMA Flow Diagram [file 12888_2023_4756_MOESM1_ESM.docx]

**Figure S1** PRISMA Flow Diagram
