# Supplementary material for: Effect of pharmacogenomics testing guiding on clinical outcomes in major depressive disorder: a systematic review and meta-analysis of RCT
Source: BMC Psychiatry. 2023 May 12;23:334. doi: 10.1186/s12888-023-04756-2 (PMC10176803; doi:10.1186/s12888-023-04756-2)
Supplement: Supplementary file 2 — Supplementary Material 2 Figure S2. Summary of Risk Assessment Using the Cochrane Collaboration’s Tool For Assessing the Risk of Bias [file 12888_2023_4756_MOESM2_ESM.docx]

**Figure S2** Summary of risk assessment using the Cochrane Collaboration’s tool for assessing the risk of bias
