# Supplementary material for: Effect of pharmacogenomics testing guiding on clinical outcomes in major depressive disorder: a systematic review and meta-analysis of RCT
Source: BMC Psychiatry. 2023 May 12;23:334. doi: 10.1186/s12888-023-04756-2 (PMC10176803; doi:10.1186/s12888-023-04756-2)
Supplement: Supplementary file 3 — Supplementary Material 3 Figure S3. Funnel plot of outcomes [file 12888_2023_4756_MOESM3_ESM.docx]

**Figure S3** Funnel plot of outcomes

A, response rate at week 4; B, response rate at week 8; C, response rate at week 12; D, response rate at week 24; E, remission rate at week 4; F, remission rate at week 8; G, remission rate at week 12; H, remission rate at week 24; I, medication congruence in 30 days
