# Supplementary material for: Effect of pharmacogenomics testing guiding on clinical outcomes in major depressive disorder: a systematic review and meta-analysis of RCT
Source: BMC Psychiatry. 2023 May 12;23:334. doi: 10.1186/s12888-023-04756-2 (PMC10176803; doi:10.1186/s12888-023-04756-2)
Supplement: Supplementary file 4 — Supplementary Material 4 Figure S4. Sensitivity Analysis of included studies [file 12888_2023_4756_MOESM4_ESM.docx]

**Figure S4** Sensitivity Analysis of included studies

A, response rate at week 4; B, response rate at week 8; C, response rate at week 12; D, response rate at week 24; E, remission rate at week 4; F, remission rate at week 8; G, remission rate at week 12; H, remission rate at week 24; I, medication congruence in 30 days; OR, odds ratio; CI, confidence interval
