# Supplementary material for: Effect of pharmacogenomics testing guiding on clinical outcomes in major depressive disorder: a systematic review and meta-analysis of RCT
Source: BMC Psychiatry. 2023 May 12;23:334. doi: 10.1186/s12888-023-04756-2 (PMC10176803; doi:10.1186/s12888-023-04756-2)

**Figure S5** Forest plot of response and remission rate comparing pharmacogenomic guided treatment versus usual care treatment when not separating data into different weeks, (A) response, (B) remission


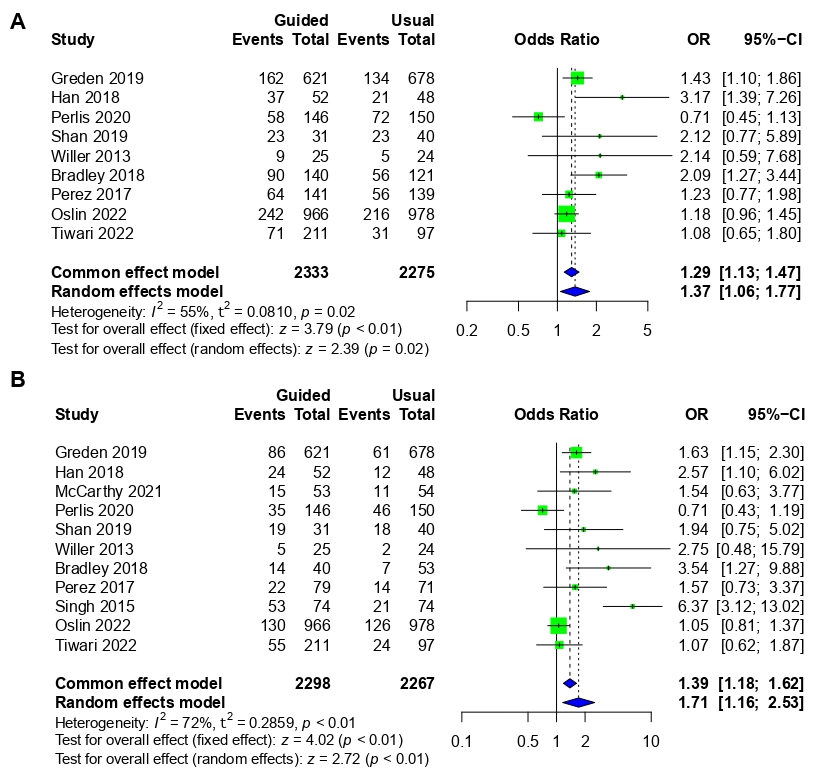

Supplement: Supplementary file 5 — Supplementary Material 5 Figure S5. Forest plot of response and remission rate comparing pharmacogenomic guided treatment versus usual care treatment when not separating data into different weeks [file 12888_2023_4756_MOESM5_ESM.docx]
