# Supplementary material for: Effect of pharmacogenomics testing guiding on clinical outcomes in major depressive disorder: a systematic review and meta-analysis of RCT
Source: BMC Psychiatry. 2023 May 12;23:334. doi: 10.1186/s12888-023-04756-2 (PMC10176803; doi:10.1186/s12888-023-04756-2)
Supplement: Supplementary file 6 — Supplementary Material 6 Table S1. Search Terms [file 12888_2023_4756_MOESM6_ESM.docx]

**Table S1** Search Terms

| PubMed |
| --- |
| ((pharmacogenomics[Title/Abstract]) OR (pharmacogenetics[Title/Abstract])) AND ((antidepressant[Title/Abstract]) OR (depression[Title/Abstract]) OR (depressive[Title/Abstract])) AND ((trial[Title/Abstract]) OR (cohort[Title/Abstract]) OR (guided[Title/Abstract]) OR (study[Title/Abstract])) |
| Embase |
| #1 'pharmacogenetic testing'/exp  #2 pharmacogenetic  #3 pharmacogenomic  #4 'antidepressant agent'/exp  #5 cohort  #6 trial  #7 guided  #8 study  #9 #1 OR #2 OR #3  #10 #5 OR #6 OR #7 OR #8  #11 #4 AND #9 AND #10 |
| Cochrane library of clinical trials |
| #1 Mesh descriptor: [Pharmacogenetics] explode all trees  #2 Mesh descriptor: [Antidepressive Agents] explode all trees  #3 #1 AND #2 |
